# Dual Reproductive Cell-Specific Promoter-Mediated Split-Cre/LoxP System Suitable for Exogenous Gene Deletion in Hybrid Progeny of Transgenic *Arabidopsis*

**DOI:** 10.3390/ijms22105080

**Published:** 2021-05-11

**Authors:** Chen Yang, Jia Ge, Xiaokang Fu, Keming Luo, Changzheng Xu

**Affiliations:** Chongqing Key Laboratory of Plant Resource Conservation and Germplasm Innovation, Key Laboratory of Eco-Environments of Three Gorges Reservoir Region, Ministry of Education, School of Life Sciences, Southwest University, Chongqing 400715, China; carolmecc@126.com (C.Y.); cqugj_2010@163.com (J.G.); fuxk92@163.com (X.F.)

**Keywords:** *Arabidopsis*, biosafety, Cre/LoxP, hybrid, reproductive cell specificity

## Abstract

Genetically modified (GM) crops possess some superior characteristics, such as high yield and insect resistance, but their biosafety has aroused broad public concern. Some genetic engineering technologies have recently been proposed to remove exogenous genes from GM crops. Few approaches have been applied to maintain advantageous traits, but excising exogenous genes in seeds or fruits from these hybrid crops has led to the generation of harvested food without exogenous genes. In a previous study, split-Cre mediated by split intein could recombine its structure and restore recombination activity in hybrid plants. In the current study, the recombination efficiency of split-Cre under the control of ovule-specific or pollen-specific promoters was validated by hybridization of transgenic *Arabidopsis* containing the improved expression vectors. In these vectors, all exogenous genes were flanked by two *loxP* sites, including promoters, resistance genes, reporter genes, and split-*Cre* genes linked to the reporter genes via LP4/2A. A gene deletion system was designed in which *NCre* was driven by *proDD45*, and *CCre* was driven by *proACA9* and *proDLL*. Transgenic lines containing NCre were used as paternal lines to hybridize with transgenic lines containing CCre. Because this hybridization method results in no co-expression of the *NCre* and *CCre* genes controlled by reproduction-specific promoters in the F1 progeny, the desirable characteristics could be retained. After self-crossing in F1 progeny, the expression level and protein activity of reporter genes were detected, and confirmed that recombination of split-Cre had occurred and the exogenous genes were partially deleted. The gene deletion efficiency represented by the quantitative measurements of GUS enzyme activity was over 59%, with the highest efficiency of 73% among variable hybrid combinations. Thus, in the present study a novel dual reproductive cell-specific promoter-mediated gene deletion system was developed that has the potential to take advantage of the merits of GM crops while alleviating biosafety concerns.

## 1. Introduction

To improve the characteristics of agricultural products, new plant breeding techniques have been developed to produce genetically modified (GM) crops with advantageous traits, such as high yield, insect resistance and abiotic stress resistance [1,2]. Facilitated by modern biotechnology, in 2019 approximately 190.4 million hectares of biotech crops were planted in 29 countries, according to the International Service for the Acquisition of Agri-Biotech Applications [3]. Traditional genetic engineering techniques modify target genomes by inserting DNA elements stably, but the escape of exogenous genes from GM crops may cause damage to the original ecological environment [2,4]. This has raised public concern about the biosafety of GM crops [5,6], and many research laboratories have begun to resolve such issues by improving biotechnology [5,7].

The strategies that have been reported to resolve GM crop biosafety issues can be divided into four types: prevention of gene escape, screening of safety marker genes, gene editing technology, and gene deletion technology. Chloroplast transformation is an effective method for preventing exogenous gene escape because chloroplast genes are inherited from the maternal line and do not escape from pollen [8], but transgenic chloroplast technology is limited because many species have biparental chloroplast inheritance. Zinc-finger nuclease, and TALEN- and CRISPR-mediated gene editing technology can accurately edit the target gene sites of crops, but new exogenous genes can be introduced into crops [9,10,11,12]. Gene deletion technologies such as co-transformation, site-specific recombinase systems, transposon positioning, and homologous recombination are often used to delete exogenous genes in transgenic plants [13,14,15,16,17].

The site-specific recombinase system mainly consists of recombinase and its recognized DNA sequence [17]. The Cre/LoxP, FLP/FRT, and R/RS systems are currently widely used for gene deletion [18,19,20]. Because of its simplicity and high efficiency, the Cre/LoxP system plays an important role in the whole recombinase family [18]. Cre recombinase, which is composed of 343 amino acids, originates from bacteriophage P1 and mainly facilitates site-specific recombination by recognizing *loxP* sites [21,22]. The arrangement of asymmetric spacer sequences in *loxP* sites determines the different modes of action of the Cre/LoxP system [23]. When two *loxP* sites are incorporated into the genomic locus in the same orientation, the exogenous genes flanked by them will be deleted by Cre-mediated recombination [22]. Based on this, the Cre/LoxP recombinase system has been successfully used for gene deletion in a variety of species of animals and plants [24,25,26,27].

In higher plants, the traditional method for deleting exogenous genes is to hybridize transgenic lines containing CRE protein and exogenous genes between two *loxP* sites [28,29]. Luo et al. [30] constructed a new “gene-deletor” system based on a Cre/LoxP and FLP/FRT site-specific recombinase system to accurately remove all functional transgenes. This system is not suitable for hybrid crops because all the gene components are located in a single vector. For application in hybrid crops, Wen et al. [31,32] and Ge et al. [31,32] generated a new “split-Cre” gene deletion system. By comparison, the N-terminal and C-terminal of the Cre protein that was split at the Asp232/Asp233 site (at the 866 bp site of its coding sequence) had high recombination efficiency, and then were expressed independently in two transgenic lines. Although this system was feasible in hybrid *Arabidopsis*, the exogenous genes were not completely deleted, and the efficiency of recombination could not be calculated [31,32].

In the current study, the split-Cre gene deletion system was improved. First, all the gene elements were flanked by two repeat *loxP* sites. The known reproductive cell-specific promoters, including the ovule-specific promoter *proDD45* [33], the pollen-specific promoter *proACA9* [34], and *proDLL* [35], were used to control spatial and temporal expression of *NCre/CCre* to retain the advantageous characteristics of GM crops but negate the presence of exogenous genes in seeds or fruits. The expression of reporter genes was used as an indicator of recombinase activity. To avoid interfering with the recombination activity of the Cre protein, the LP4/2A leader peptide with an intervening sequence (“linker peptide”) originating from a natural polyprotein in seed of *Impatiens balsamina* was used to connect *NCre/CCre* with the reporter gene [36]. By way of the unique design of the combination of hybridization, the F1 progeny only express exogenous genes in the vegetative growth stage, and then the exogenous genes are deleted in the reproductive growth stage. The analysis of exogenous gene deletion efficiency in F2 hybrid progeny proved that the reproductive cell-specific promoter-mediated split-Cre gene deletion system could knock out the exogenous genes in the seeds of F1 progeny. In the current study, a novel gene deletion system with the potential to retain the advantageous characteristics of transgenic plants while precluding the retention of exogenous genes in fruits and seeds was developed.

## 2. Results

### 2.1. Construction of Vectors Containing Split-Cre Recombinase Driven by Reproductive Cell-Specific Promoters

**Figure 1:**
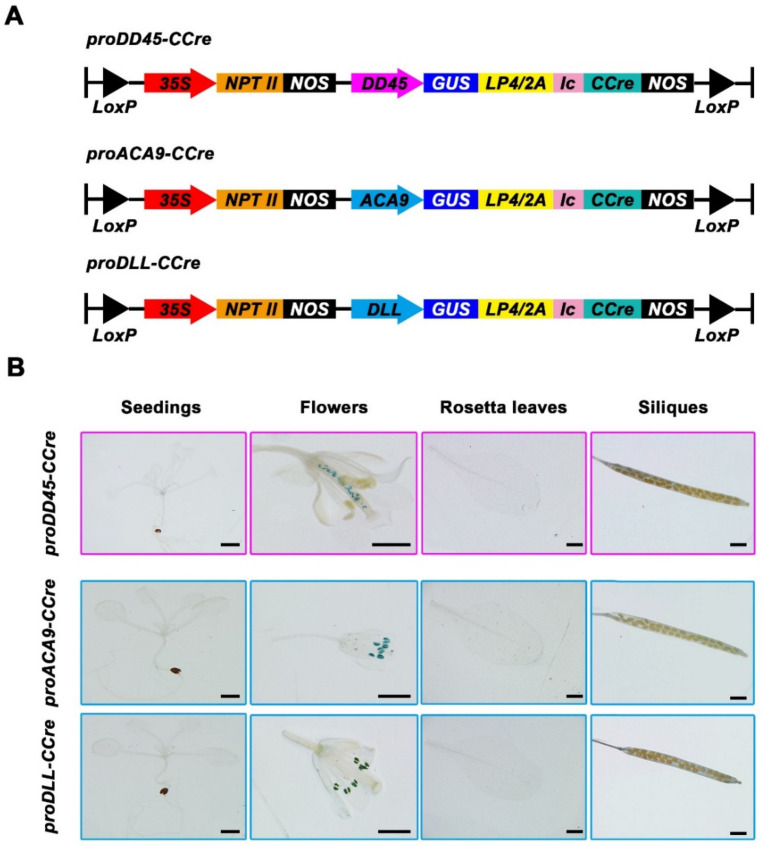
Identification of reproductive cell specific promoters. (**A**) Schematic illustration of the binary expression vectors which contained split-*Cre* driven by reproductive cell specific promoters. Gene cassettes used in the split-*Cre* vector named *proDD45-CCre*, *proACA9-CCre*, and *proDLL-CCre*. The resistance gene *NPTII* was driven by the *CaMV35S* promoter. *Nos*, 30-untranslated sequence from the *Agrobacterium NOS* gene. *LP4/2A*, connecting peptide; *Ic*, the C terminus of intein. The *CCre* fused with the reporter gene *GUS* by *LP4/2A* was driven by the promoter of *DD45*, *ACA9*, and *DLL*, respectively. (**B**) Histochemical analysis of GUS activity of different tissues in *proDD45-CCre*, *proACA9-CCre*, and *proDLL-CCre* transgenic *Arabidopsis*. Bars: 1 mm.

In previous research, the recombination activity of split-Cre could be obtained when both NCre and CCre were supplied [31], and the recombination activity of split-*Cre* linked to split-intein (the *DnaE* gene of *Synechocystis* sp.) was higher in the hybrid progeny of transgenic *Arabidopsis* when Cre recombinase was split on Asp232/Asp233 (866 bp) cleavage points [32]. Notably however, the exogenous genes in this gene deletion system could not be totally excised. To achieve the complete deletion of exogenous genes, a series of vectors used for split-*Cre* complementation were constructed. All the components, including promoters, reporter genes, split-*Cre* genes, and other exogenous genes, were flanked by two *loxP* sites in the same direction, and the C-terminal (*Ic*) fragments of intein linked to *CCre* were fused with the *GUS* reporter gene via a linker LP4/2A [36] (Figure 1A).

To ensure split-Cre-mediated gene deletion only occurred in reproductive growth stages and retain the advantageous characteristics in the hybrid transgenic crops, the *CCre* gene fused with the *GUS* gene was controlled by the ovule-specific promoter *proDD45* [33], the pollen-specific promoter *proACA9* [34], and *proDLL* [35] (Figure 1A). Expression levels of the *CCre* gene in wild-type and transgenic plants containing *proDD45-CCre*, *proACA9-CCre*, and *proDLL-CCre* were detected via RT-qPCR. Compared with the wild-type, *CCre* gene expression was higher in transgenic plants (Supplementary Materials Figure S1). GUS histochemical staining was conducted in different tissues to verify the tissue specificity of promoters. GUS signals were only detected in pollen grains of *proACA9-CCre* and *proDLL-CCre* transgenic plants, and the GUS signal was exclusively restricted to the ovules of transgenic plants containing the *proDD45-CCre* vector (Figure 1B).

### 2.2. Validation of the Recombination Efficiency of Split-Cre Recombinase Driven by an Ovule-Specific Promoter

**Figure 2:**
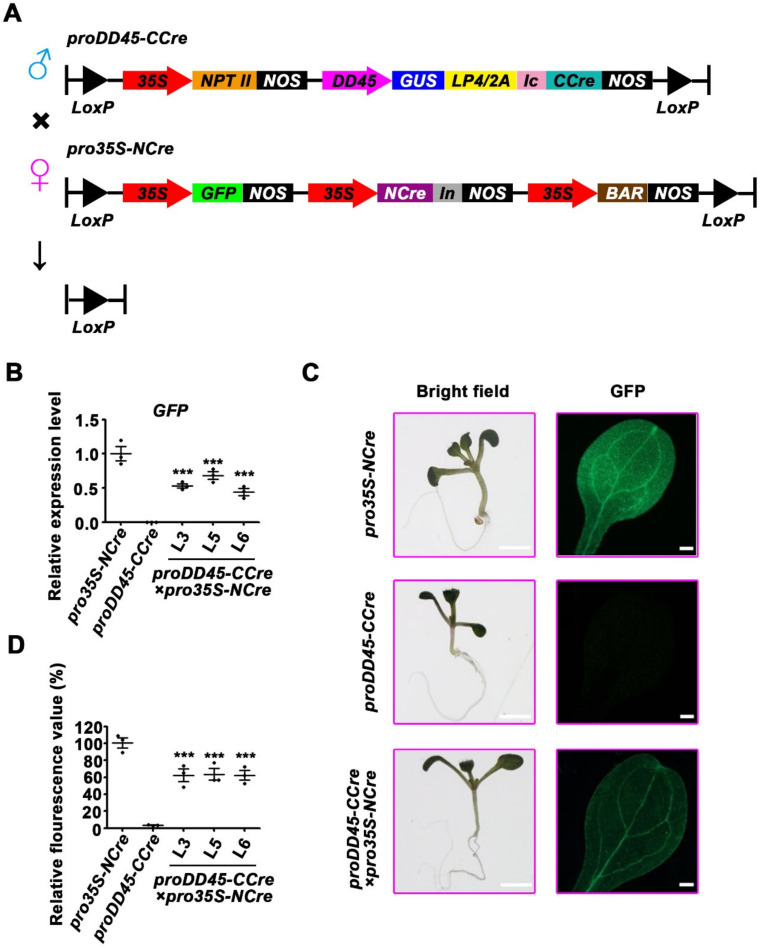
Analysis of the recombination activity of split-Cre controlled by ovule-specific promoter in F2 progeny of hybridization *Arabidopsis*. (**A**) Schematic diagram of plant expression vectors used for hybridization. The transgenic *Arabidopsis* harboring the binary vector *pro35S-NCre* was used as the maternal lines, while that containing the *proDD45-CCre* was used as the paternal lines. *In*, the N terminus of intein. (**B**) The expression level of the reporter gene *GFP* in the 10-day-old F2 progeny of *proDD45-CCre*×*pro35S-NCre* hybridization *Arabidopsis.* (**C**,**D**) The observation and valuation of the GFP fluorescence of first-pair rosette leaves in the 10-day-old F2 progeny seedlings of *proDD45-CCre*×*pro35S-NCre* transgenic *Arabidopsis.* Bars: 1 mm (for bright field); bars: 20 μm (for microscope). Error bars represent ±SEM of three biological replicates. Asterisks indicate significant differences using Student’s *t*-test (*** *p* < 0.001).

To determine whether the *Ic-CCre* mediated by the ovule-specific promoter *proDD45* could recover its recombination activity in progeny of hybridized transgenic *Arabidopsis*, the expression vector *pro35S-NCre* was constructed (Figure 2A). In this vector, the expression of *NCre-In* was controlled by the constitutive *CaMV 35S* promoter, and all the gene components were flanked by two repeat *loxP* sites (Figure 2A). *Pro35S-NCre* homozygous transgenic plants with a single copy were treated as the maternal lines to hybridize with the three homozygous transgenic lines harboring the vector *proDD45-CCre* (Figure 2A). Due to this hybridization formation, the recombination activity should be absent in F1 progeny, which benefits the retention of the superior characteristics.

**Figure 3:**
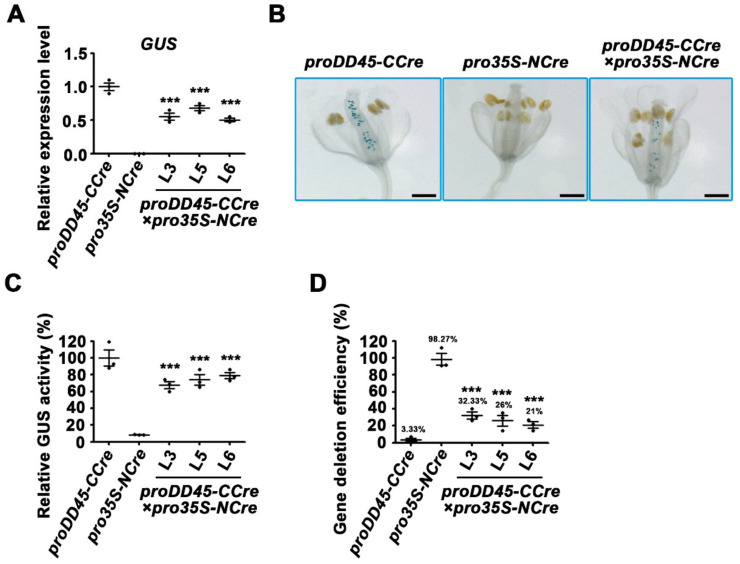
The recombination efficiency of split-Cre in F2 progeny of hybridization *Arabidopsis* when CCre protein was controlled by ovule-specific promoter. (**A**) The RT-qPCR analysis of *GUS* gene in flowers of *proDD45-CCre*×*pro35S-NCre* hybridization *Arabidopsis*. (**B,C**) Histochemical and quantitative analysis of GUS activity of flowers in F2 progeny of *proDD45-CCre*×*pro35s-NCre*. Bar: 1 mm. (**D**) Recombination efficiency in the transgenic *Arabidopsis* determined by GUS activity. Error bars represent ±SEM of three biological replicates. Asterisks indicate significant differences using Student’s *t*-test (*** *p* < 0.001).

F2 progeny lines were obtained by self-crossing the F1 progeny lines. The death ratio of F2 progeny grown on the medium containing two kinds of antibiotics was in accordance with Mendel’s laws of independent gene assortment and free recombination, which indicated that the antibiotic-sensitive lines were all produced by free recombination of genes rather than the foreign genes being excised completely by recombination of the split-Cre (Supplementary Materials Table S1). To test whether the activity of split-Cre was recovered and the exogenous genes were excised partially, RT-qPCR was used to analyze the expression levels of resistance genes (*NPTII* and *BAR*) and recombinase genes (*NCre* and *CCre*) in F2 progeny lines. Compared with the parent lines, all the genes detected were decreased dramatically (Supplementary Materials Figure S2). The expression level of the *GFP* reporter gene and the strength of GFP fluorescence were also markedly lower than they were in the maternal lines (Figure 2B–D). Compared with the paternal lines, transcript level of *GUS* reporter gene was also lower in the F2 progeny lines (Figure 3A). GUS activity was measured to determine the efficiency of gene deletion. The above results revealed that the split-*Cre* controlled by ovule-specific promoter can achieve recombination via hybridization, resulting in some foreign genes being deleted in F2 progeny (Figure 3B–D).

### 2.3. Recombination Activity Verification of Split-Cre Recombinase Controlled by Pollen-Specific Promoters

**Figure 4:**
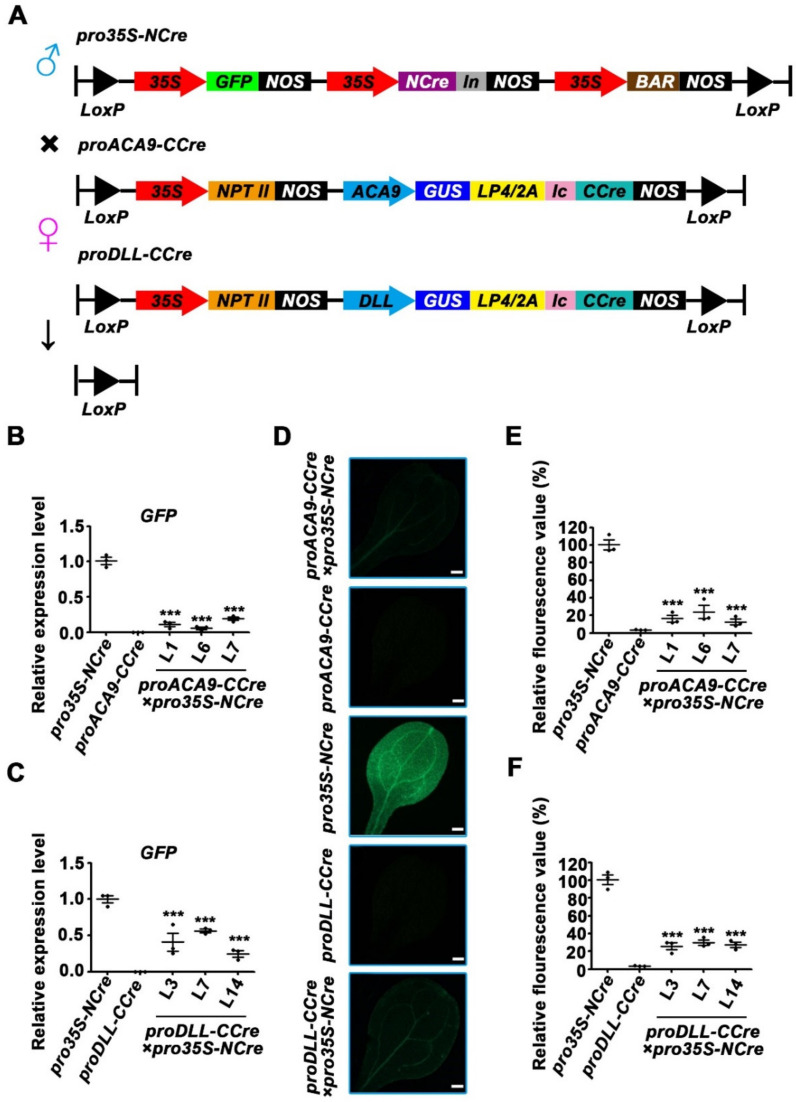
Validation of the recombination activity of split-Cre when the *CCre* gene was controlled by pollen-specific promoter. (**A**) Schematic diagram of the expression vectors containing split-Cre mediated by *CaMV35S* and pollen-specific promoters. Transgenic lines *proDLL-CCre* and *proACA9-CCre* were used as the maternal lines, whereas the lines transformed with the *pro35S-NCre* were used as the paternal lines. (**B,C**) Expression analysis of the *GFP* reporter gene in the 10-day-old F2 progeny seedlings of *proACA9-CCre*×*pro35S-NCre* and *proDLL-CCre*×*pro35S-NCre* transgenic *Arabidopsis.* (**D**) Observation of GFP fluorescence of the first-pair rosette leaves in 10-day-old F2 progeny seedlings. Bars: 1 mm (for bright field); bars: 200 μm (for microscope). (**E,F**) The measurement of the GFP value of the first-pair rosette leaves in 10-day-old F2 progeny seedlings. Error bars represent ±SEM of three biological replicates. Asterisks indicate significant differences using Student’s *t*-test (*** *p* < 0.001).

**Figure 5:**
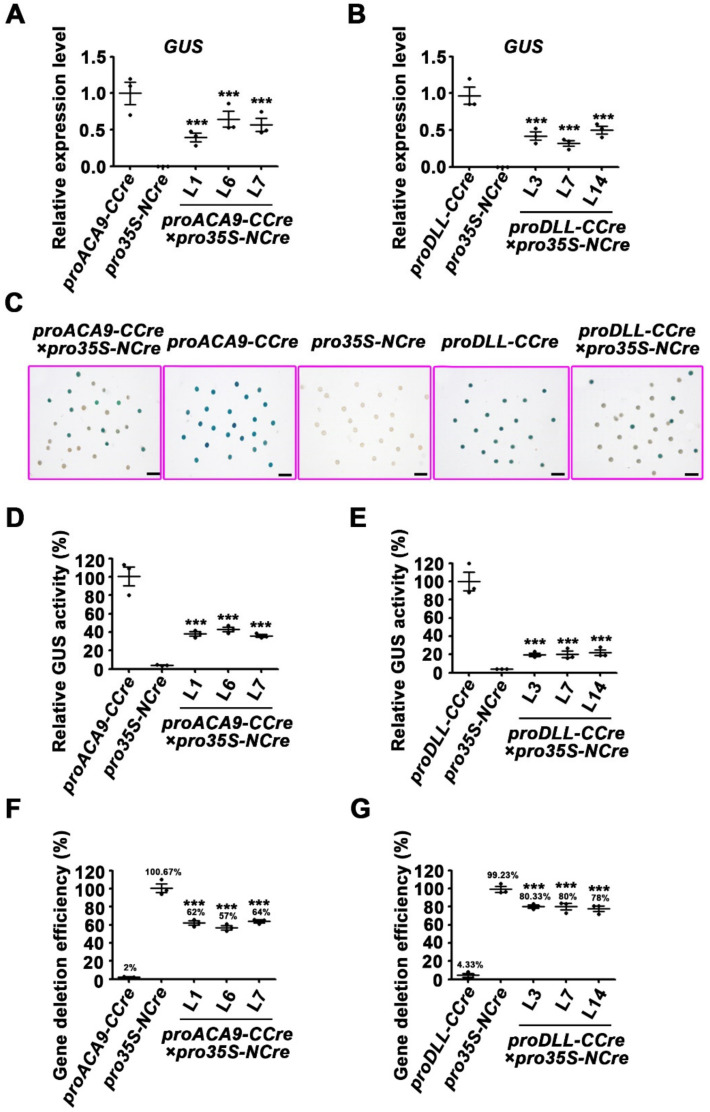
The recombination efficiency of split-Cre mediated by pollen-specific promoters in F2 progeny of hybridization *Arabidopsis.* (**A**,**B**) The RT-qPCR analysis of the *GUS* reporter gene in 10-day-old *proACA9-CCre*×*pro35S-NCre* and *proDLL-CCre*×*pro35S-NCre* transgenic *Arabidopsis* seedlings. (**C**) GUS staining of various pollen grains from the F2 progeny of pro*ACA9-CCre*×*pro35S-NCre* and *proDLL-CCre*×*pro35S-NCre*. The transformants containing the vector *proDLL-CCre* or *proACA9-CCre* were used as the positive control, and *pro35S-NCre* was used as the negative control. Bars: 200 μm. (**D**,**E**). The GUS activity of various pollen grains from the F2 progeny of pro*ACA9-CCre*×*pro35S-NCre* and *proDLL-CCre*×*pro35S-NCre*, respectively. (**F**,**G**) Recombination efficiency of these two hybridization combinations in the transgenic *Arabidopsis* determined by GUS activity of various pollen grains from the F2 progeny. Error bars represent ±SEM of three biological replicates. Asterisks indicate significant differences using Student’s *t*-test (*** *p* < 0.001).

Experiments were conducted to further determine whether the *CCre* gene controlled by the pollen-specific promoters *proDLL* and *proACA9* could be co-expressed with the *NCre* gene driven by the constitutive *CaMV 35S* promoter in hybrid progeny of transgenic plants (Figure 4A). Transgenic plants harboring the *proACA9-CCre* or *proDLL-CCre* were treated as the maternal lines and hybridized with male paternal lines transformed with *pro35S-NCre*. The F2 progeny lines were obtained by self-crossing F1s. The segregation ratios of F2 seedlings grown normally on medium containing kanamycin and phosphinothricin were approximately 0.4375, which is in accordance with the expected Mendelian inheritance ratio. These statistical data suggest that the exogenous genes were not excised completely in seedlings (Supplementary Materials Table S2). However, the results of RT-qPCR revealed that the expression levels of both resistance genes and split-*Cre* genes were lower in the living seedlings (Supplementary Materials Figure S3). It is likely that the Cre protein recovered its activity and then deleted the genes flanked by the repeat *loxP* sites in some cells. To further confirm that gene deletion occurred in the F2 progeny, transcription levels and protein activity of the reporter genes were analyzed. Compared with the parent lines, GFP and GUS activity were much lower in F2 progeny (Figure 4B–F and Figure 5A–E). Statistical analysis of gene deletion efficiency by GUS activity indicated that the split-*Cre* genes driven by pollen-specific promoters could achieve protein recombination, and mediated gene deletion (Figure 5F,G).

### 2.4. Analysis of Gene Deletion Efficiency in the Dual Reproductive Cell-Specific Promoter-Mediated Split-Cre/LoxP System

**Figure 6:**
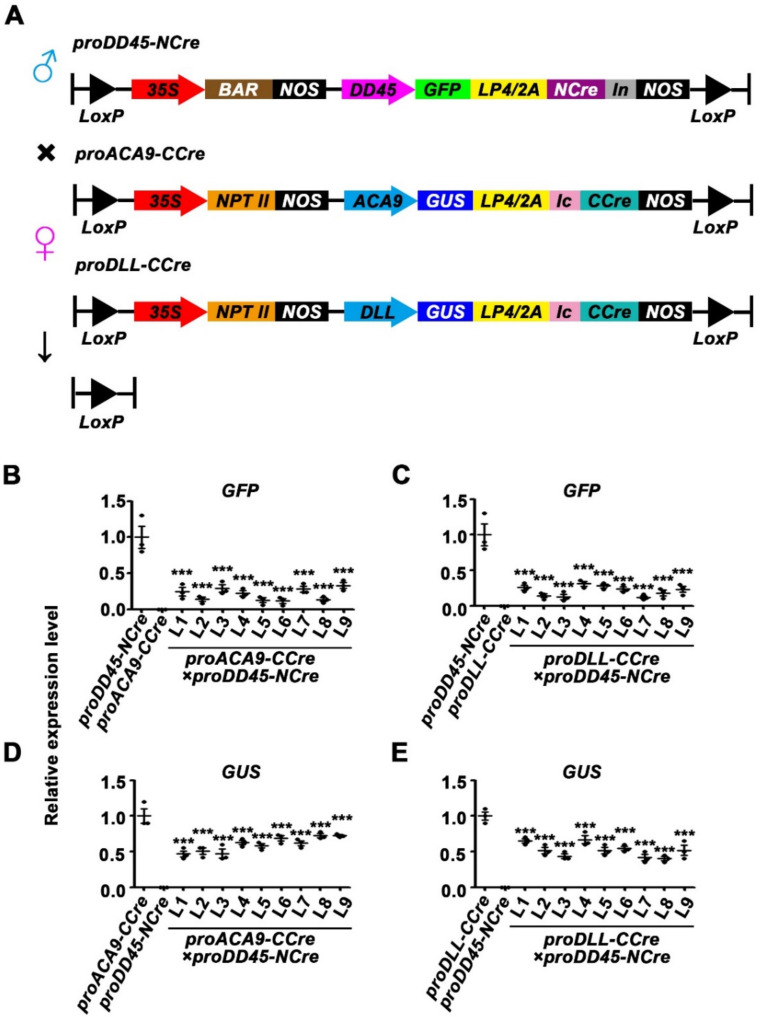
Transcriptional level of exogenous reporter genes in the dual reproductive cell-specific promoter-mediated split-Cre/LoxP system. (**A**) Schematic diagram of dual reproductive cell-specific promoter-mediated plant expression vectors used for hybridization. Transgenic lines containing *proDD45-NCre* were used as the paternal lines, and the *proACA9-CCre* and *proDLL-CCre* were used as the maternal lines. (**B**–**E**) Expression level of *GFP* and *GUS* reporter genes in 10-day-old F2 progeny seedlings of *proACA9-CCre*×*proDD45-NCre* and *proDLL-CCre*×*proDD45-NCre* transgenic *Arabidopsis.* Error bars represent ±SEM of three biological replicates. Asterisks indicate significant differences using Student’s *t*-test (*** *p* < 0.001).

**Figure 7:**
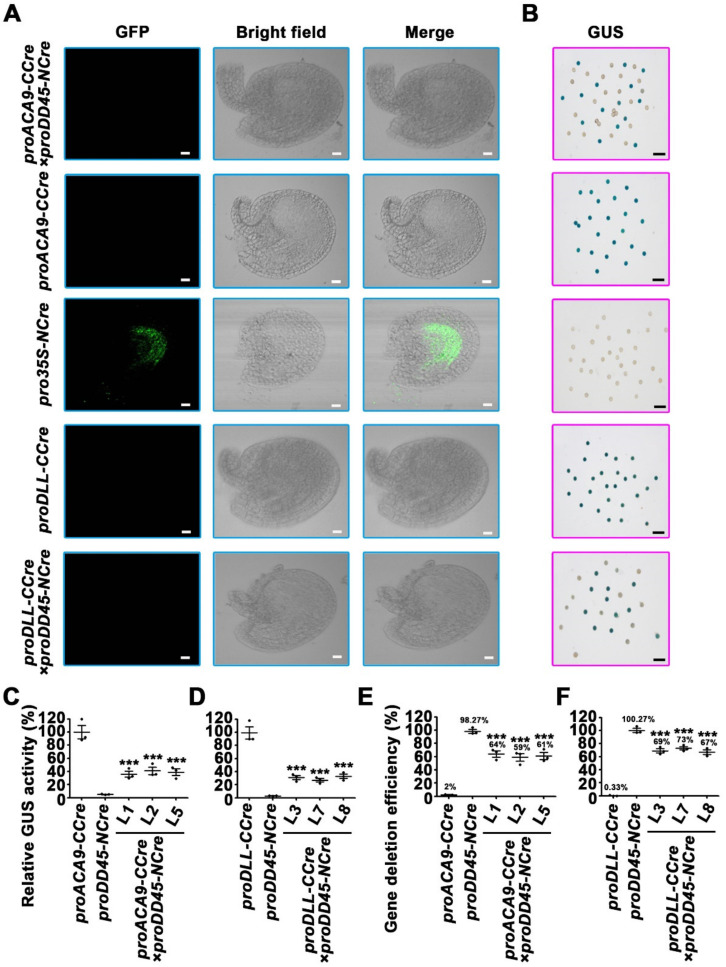
The exogenous gene deletion efficiency of the split-Cre/LoxP recombinase system mediated by dual reproductive cell-specific promoters. (**A**) The GFP fluorescence observation of ovules in F2 progeny of *proACA9-CCre*×*proDD45-NCre* and *proDLL-CCre*×*proDD45-NCre* hybridization *Arabidopsis*. Bars: 20 μm. (**B**) GUS histochemical staining of pollen grains in hybridization progeny mediated by the dual reproductive cell-specific promoter-mediated system. Bars: 200 μm. (**C**,**D**) Quantification of GUS activity of pollen grains in F2 progeny. (**E**,**F**) The gene deletion efficiency in F2 progeny of *proACA9-CCre*×*proDD45-NCre* and *proDLL-CCre*×*proDD45-NCre.* Error bars represent ±SEM of three biological replicates. Asterisks indicate significant differences using Student’s *t*-test (*** *p* < 0.001).

**Figure 8:**
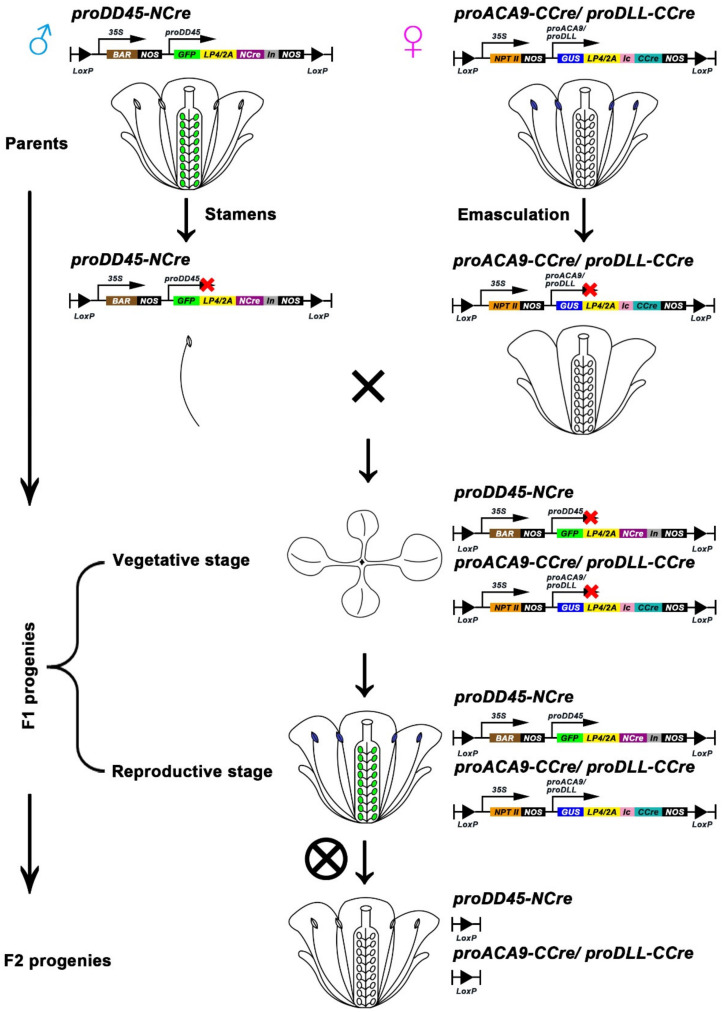
The hybridization model of the dual reproductive cell-specific promoter-mediated split-Cre/LoxP system. After emasculation, the *proACA9/DLL-CCre* transgenic lines were pollinated with the pollen of *proDD45-NCre* transgenic lines. The F1 progeny of hybridization plants showed “excellent characters”, represented by antibiotic resistance in vegetative stage. In addition, gene deletion occurred in the seeds or fruits of F1 progeny after self-crossing, and then the exogenous gene-free F2 progeny was generated.

The results described above revealed that the *CCre* gene driven by an ovule-specific promoter or a pollen-specific promoter co-expressed with *NCre* controlled by the constitutive *CaMV 35S* promoter led to complementation of the Cre protein, and recovery of its activity. To retain advantageous traits in hybrid progeny but harvest seeds or fruits without exogenous genes, the Cre/LoxP gene deletion system was developed, in which the expression of *NCre* and *CCre* genes were both controlled by reproductive promoters (Figure 6A). Tissue-specific expression of *proDD45* was verified via GFP observation (Supplementary Materials Figure S4). Transgenic plants harboring *NCre* controlled by the ovule-specific promoter *proDD45* were used as paternal lines, and transgenic ones harboring *CCre* driven by the pollen-specific promoters *proDLL* and *proACA9* were used as maternal lines (Figure 6A). This hybridization strategy resulted in the recombination of Cre protein after self-crossing F1 hybrid progeny. Therefore, we focused on F2 progeny. Compared with the parent lines, the transcription levels of all exogenous genes including split-*Cre* genes, reporter genes, and resistance genes were significantly down-regulated in the F2 progeny of the two hybrid combinations (Figure 6B–E and Supplementary Materials Figure S5). Three of the nine transgenic lines were randomly selected as representatives to analyze the protein activity of the reporter genes. Barely any GFP signal was observed in F2 progeny, and most pollen grains in F2 progeny did not turn blue after GUS histochemical staining (Figure 7A,B). More detailed quantitative measurements of GUS enzyme activity were also concordant with these observations (Figure 7C,D), suggesting that the dual reproductive cell-specific promoter-mediated split-Cre/LoxP system can delete exogenous genes and achieve high deletion efficiency (Figure 7E,F), although the exogenous genes were not completely deleted (Figure 7E,F and Supplementary Materials Table S3). In the present study, a novel split-Cre/LoxP gene deletion system was developed that is mediated by dual reproductive cell-specific promoters, and has the potential to generate GM crops with retained advantageous traits that produce seeds or fruits without exogenous genes (Figure 8).

## 3. Discussion

Although transgenic technologies have facilitated the generation of new crop strains with some advantageous characteristics, the potential problem of biosafety has aroused extensive attention [2,5]. In the current study, a novel dual reproductive cell-specific promoter-mediated gene deletion system based on the Cre/LoxP recombinase system was developed. In the hybrid progeny generated using this system, the advantageous characteristics are retained, but the exogenous genes are deleted from the seeds and fruits of these progeny. This gene deletion system constitutes a new strategy for addressing the biosafety concerns pertaining to GM crops, without losing their associated advantages.

In previous studies, Cre recombinase has been reportedly split into two complementation polypeptides, and recombination activity could be restored in vivo [37,38,39]. The split-Cre gene deletion system has been improved and used in hybrid crops [32]. Notably, the existing gene deletion system has not been able to completely delete exogenous genes. Most gene deletion events occur in the vegetative growth stage in the progeny of hybrid crops, which can result in the advantageous traits of the GM crops being lost. To better utilize the Cre/LoxP gene deletion system, two aspects were improved in the new deletion system described herein. One is that all exogenous genes, including vector sequences, promoter sequences, resistance genes, reporter genes, and split-*Cre* genes, are flanked by two *loxP* sites, which means they can be completely deleted via the recombination of Cre. The other is that *NCre* and *CCre* genes are respectively driven by an ovule-specific promoter and a pollen-specific promoter. Moreover, transgenic lines expressing *NCre* specifically in ovule cells were used as paternal lines to hybridize with the maternal lines in which *CCre* was expressed exclusively in pollen grains. Due to this hybridization pattern, gene deletion did not occur in the vegetative growth stage of F1 hybrid progeny until the plants grew into the reproductive growth stage.

The deletion of exogenous genes can be identified by the expression levels of reporter genes and the transcription levels and protein activity of reporter genes in F2 hybrid progeny. Gene deletion efficiency can be determined via GUS activity, and can be calculated in F2 hybrid progeny (the seeds of F1 hybrid progeny). Based on these results, the deletion of exogenous genes in F2 hybrid progeny was confirmed in the present study (Figure 6 and Figure 7). The efficiency of gene deletion was not 100%, and this may be because *NCre* and *CCre* were also flanked by two *loxP* sites and thus deleted as exogenous genes (Figure 7E,F). The deletion of *NCre* and *CCre* genes limits the efficiency of a split-*Cre*-mediated gene deletion system, resulting in some exogenous genes remaining. In addition, in the current study, differential assembly of reproductive cell-specific promoters led to differential gene deletion efficiency. For example, the deletion efficiency of the hybrid combination mediated by the *DLL* promoter was higher than that of the hybrid combination mediated by the *ACA9* promoter, even though both *proDLL* and pro*ACA9* were pollen-specific promoters (Figure 5F,G and Figure 7E,F). There was no significant correlation between deletion efficiency and promoter expression intensity, but not all of the hybrid combinations mediated by the stronger promoter *CaMV 35S* were more efficient deletion systems. This indicates that the suitable assembly of two promoters—including similar expression levels and the accuracy of spatiotemporal expression of promoters—were more important for improving deletion efficiency (Figure 3C,D and Figure 7E,F). Therefore, screening suitable native reproductive promoters and/or constructing artificial promoters will be key aspects of further research.

Despite the so-called “green revolution” that began in the 1960s, based on current crop production growth trends, the required global demand will not be met by 2050 because crop yields can be limited by many factors, such as pests and damage to the environment [40,41,42]. Plant genetic transformation technology has brought new traits to crops, enabling them to resist these stresses to an extent, but it has also caused consumers to worry about biosafety [5,43]. The split-*Cre* gene deletion system controlled by reproductive promoters described herein has been verified as functional in *A. thaliana*. If the deletion efficiency of the system can be improved, and it can be extended to major staple grain crops, it may become an effective means of addressing post-green revolution challenges.

## 4. Materials and Methods

### 4.1. Vectors Construction

The *pro35S-NCre866-In-nos* fusion segments were excised from *pCA-NCre-In* [32] with *SacI* and ligated into the same sites of the corresponding *pCA-NOS* to product the vectors *pCA-NOS-NCre-In*. The gene fragments of *GFP*, *NOS,* and *BAR* were amplified with specific primers (listed in Supplementary Materials Table S4). These PCR products were firstly cloned into *pCXSN* [44], respectively. Then the gene fragments of *pro35S-GFP* and *pro35S-BAR-NOS* were inserted into the *pCA-NOS-NCre-In* as the *pro35S-NCre*.

Gene-specific primers (Supplementary Materials Table S4), *proDD45*, *proDLL*, and *proACA9* were isolated from the genomic DNA of *Arabidopsis*. Based on the *pro35S-NCre*, the gene fragments *GFP-LP4* and *2A-NCre-In* were amplified with specific primers and then cloned to *pMD19-T* (Takara, Dalian, China), resulting in *pMD19-GFP-LP4/2A-NCre-In*. The fragment *pro35S-BAR-NOS* was first cloned to *pCXSN* and then ligated to *pCA-NOS* by *Hin*dIII and *Eco*RI as the vector *pLML-NOS-BAR*. By *Avr*II and *Sac*I, the intact fragment of *GFP-LP4/2A-NCre-In* were inserted into *pLML-NOS-BAR*, and *proDD45* was finally ligated with *Spe*1as the final vector *proDD45-NCre*.

The fragment of *GUS-LP4* was amplified from the vector *pCMBA1305.1* with *GUS-F*, *GUS-LP4-R1,* and *GUS-F1*, *LP4-R2* primer pairs. The *2A/Ic-CCre* fusion fragment was isolated from the *pCA-IC-CCre* [32] with primers listed in Supplementary Materials Table S4 and cloned to *pMD19* via *Avr*II and *Sac*I; *GUS-LPA/2A-Ic-CCre* was inserted into the *pLML-NOS-NPTII*, which was conducted by ligating *NOS-NPTII* to *pLML* as *pLML-NOS-NPTII-CCre*. Finally, *proDD45*, *proDLL*, and *proACA9* were cloned to it, respectively, as the expression binary vectors *proDD45-CCre*, *proDLL-CCre*, and *proACA9-CCre*.

### 4.2. Genetic Transformation and Plant Materials

*Arabidopsis thaliana* ecotypes Columbia (Col-0) was used as wild-type and for generating various transgenic lines. Plants were germinated on MS medium for 10 days and then planted in soil under the condition of 23 °C room temperature, 16 h 10,000 lux light and 8 h dark cycle. Genetic transformation of *Arabidopsis* was conducted by the *Agrobacterium tumefaciens*-mediated floral dip method as described previously [45]. The homozygous transgenic plants with a single copy were used for further experiments and hybridization as the Supplementary Materials Table S5.

### 4.3. RNA Extraction and Quantitative RT-PCR

Total RNA from different tissues of 10-day-old *Arabidopsis* was extracted by RNA RNeasy Plant Mini Kit (Qiagen, Maryland, Germany). Followed by removal of genomic DNA with RNase-free DNase (Takara, Dalian, China), cDNA was obtained with the PrimeScript RT reagent Kit (Takara, Dalian, China). The SYBR Green PCR master mix kit (Takara, Dalian, China) was used in the RT-qPCR reaction, performed by the TP700 Real-Time PCR machine (Takara, Japan). The *UBQ* gene of *Arabidopsis* was regarded as the reference gene. Three biological replicates were performed for all of the samples and three technical replicates were carried out for each reaction. The sequences of primers used are listed in Supplementary Materials Table S4.

### 4.4. GFP fluorescence Detection

The GFP signal of *Arabidopsis* was observed by microscope (Olympus BX53, Tokyo, Japan). The ovules were then carefully split from the flowers using tweezers and fixed in a glass slide. The detection and measurement of GFP fluorescence in ovules of *Arabidopsis* was excited at 488 nm, and the emission was collected with a 505 to 530 nm bandpass filter via a confocal laser microscope (Olympus FV1200, Tokyo, Japan). The fluorescence value was quantified by the software FV10 ASW (Olympus, Tokyo, Japan).

### 4.5. Histochemical Staining

Histochemical analysis of GUS activity was performed as described previously [46]. In brief, the plant tissues were placed in GUS staining solution (0.5 M Tris, pH 7.0, and 0.1% (*v*/*v*) Triton X-100 with 1 mM with 1 mM X-Gluc (5-bromo-4-choloro-3-indolyl-b-glucuronic acid)) and incubated at 37 °C for 6 h in the dark. Chlorophyll was removed using an ethanol series: 20%, 35%, and 50% (v/v) ethanol at room temperature for 30 min each until the plant tissues became transparent. The chlorophyll-free stained plant tissues were observed under the Olympus BX53 microscope. The pollen grains were separated by sufficient vortex and collected by centrifugation for GUS staining.

### 4.6. Pollen Grains Protein Extraction

The flowers of over 100 6~8 week old *Arabidopsis* were first collected in centrifugal tubes. Pollen grains were separated by sufficient vortex for 2~5 min and then collected by centrifuge at 350× *g* for 1 min at 4 °C. Pollen grains were separated from flowers by vortex and collected by centrifuge. The pollen grains were ground until the solution turned yellow, then K-HEPES protein extraction buffer was added and kept on ice for 1 h as described previously [47]. The supernatant was collected as pollen grain protein solution, and its concentration measured by standard Bradford assay for the following GUS activity assay.

### 4.7. GUS Activity Assay

After grinding in liquid nitrogen, the total protein of flowers and pollen grains was extracted by extract buffer (0.1 M Phosphate buffer solution (PH 7.0), 0.1% SDS (Sodium dodecyl sulfate), 0.01% Triton X-100, 10 mM EDTA (Ethylene Diamine Tetraacetic Acid), and 0.01% β-Mercaptoethanol) for GUS activity analysis. Quantitative analysis of GUS activity of flowers and pollen grains was measured by monitoring cleavage of 4-methyl umbelliferyl b-D-glucuronide (MUG), the substrate of b-glucuronidase, which upon hydrolysis produced the fluorescent 4-methyl umbelliferone (4 MU), and was quantitatively assayed by a spectrophotometer (F-7000, Hitachi, Tokyo, Japan) with the 365 nm excitation and the 455 nm emission filters as described previously [48]. Protein concentrations were determined via the Bradford method.

### 4.8. Deletion Efficiency Statistics

The foreign gene deletion efficiency of F2 hybrid progeny lines was calculated by GUS activity according to the following formula:

Foreign gene deletion efficiency = (1−GUS activity of F2 hybrid progeny lines/GUS activity of parental lines) × 100%.

### 4.9. Chemicals

The *pMD19-T* vector (Takara, Dalian, China); PrimerSTAR Max DNA Polymerase (Takara, Dalian, China); the restriction endonucleases (New England Biolabs, Ipswich, MA, USA); T4 DNA Ligase (Takara, Dalian, China); MS medium (Basebio, Hangzhou, China); RNA RNeasy Plant Mini Kit (Qiagen, Maryland, Germany); RNase-free DNase (Takara, Dalian, China); PrimeScript RT reagent Kit (Takara, Dalian, China); SYBR Green PCR master mix kit (Takara, Dalian, China); X-Gluc (Aladdin, Shanghai, China); MUG (Yeasen, Shanghai, China).

## Data Availability

Not applicable.

## Supplementary Materials

The following are available online at https://www.mdpi.com/article/10.3390/ijms22105080/s1. Table S1: Statistics of antibiotic-sensitive rate in hybridization F2 progenies (*proDD45-CCre × pro35S-NCre*). Table S2: Statistics of antibiotic-sensitive rate in hybridization F2 progenies (*proACA9-CCre × pro35S-NCre* and *proDLL-CCre × pro35S-NCre*). Table S3: Statistics of antibiotic-sensitive rate in hybridization F2 progenies (*proACA9-CCre × proDD45-NCre* and *proDLL-CCre × proDD45-NCre*). Table S4: List of primers used in this study. Table S5: List of hybrid combination used in this study. Figure S1: Verification of the expression of *CCre* linked to LP4/2A controlled by reproductive cell specific promoters. Figure S2: The transcriptional level of resistance genes and split-*Cre* genes driven by ovule-specific promoter *proDD45* in F2 progeny hybridization transgenic *Arabidopsis*. Figure S3: The expression level of resistance genes and split-*Cre* genes controlled by pollen-specific promoters *proACA9* and *proDLL* in F2 progeny of hybridization transgenic *Arabidopsis*. Figure S4: Generation of the transgenic *Arabidopsis* containing *NCre* genes driven by ovule-specific promoter *proDD45* lines. Figure S5: The transcriptional level of resistance genes and split-*Cre* genes in the split-Cre/LoxP recombinase system mediated by dual reproductive cell-specific promoters.
