# Supplementary material for: Dual Reproductive Cell-Specific Promoter-Mediated Split-Cre/LoxP System Suitable for Exogenous Gene Deletion in Hybrid Progeny of Transgenic Arabidopsis
Source: Int J Mol Sci. 2021 May 11;22(10):5080. doi: 10.3390/ijms22105080 (PMC8151399; doi:10.3390/ijms22105080)
Supplement: Supplementary file 1 [file ijms-22-05080-s001.zip › Supplementary File - revised/Table S4.pdf]

Table S4. List of primers used in this study.

Primer	Sequence (5'-3')
**Gene cloning and plasmid construction**	
***GFP*-F**	**5′-gaagatcttcatgaagactaatctttttc-3′**
***GFP*-R**	**5′-gaagatcttccgggatcccgttagagttcg-3′**
***NOS*-F**	**5′-gaagatcttccgttcaaacatttggcaata-3′**
***NOS*-R**	**5′-gaagatcttcggactagtcccccgatctagtaacatag-3′**
***BAR*-F**	**5′-gaagatcttcatgagcccagaacgacgccc-3′**
***BAR* -R**	**5′-gaagatcttccgggatcccgttaaatctcggtgacgggca-3′**
***proDD45*-F**	**5′-ggactagtctttgataaatgttcctcgctgacg-3′**
***proDD45*-R**	**5′-ggactagtggaaacttgtgttagaagccatta-3′**
***proDLL*-F**	**5′-gctctagatggtttttgaggcaactcccctt-3′**
***proDLL*-R**	**5′-gctctagattttctttctttcttcacttgtt-3′**
***proACA9*-F**	**5′-ggactagtacgcaaatcttgaggctgatc-3′**
***proACA9*-R**	**5′- ggactagtcattcttccttccttcgtctc-3′**
***GFP-LP4*-F**	**5′-cgcggatccatgaagactaatctttttctctttctc-3′**
***GFP-LP4*-R**	**5′-gtagccacctcgtccgccgcgttggagagttcgtcgtgtttgtatag-3′**
***2A-NCre-In*-F**	**5′-aagcttgcgggagacgtcgagtccaaccctgggcctatgcccaagaag-3′**
***2A-NCre-In* -R**	**5′-cccaagcttgagctcctatttaattgtcccagcgtcaagtaatgg -3′**
***GUS*-F**	**5′-cgcggatccatggtagatctgagggtaaatttc-3′**
***GUS-LP4*-R1**	**5′-gtagccacctcgtccgccgcgttggacacgtgatggtgatggtgatg-3′**
***GUS*-F1**	**5′-cctaggatggtagatctgagggtaaatttc-3′**
***LP4*-R2**	**5′-caagcttaagaaggtcaaaattcaacagctgggtagccacctcgtccgc-3′**
***2A/Ic-CCre*-F**	**5′-aagcttgcgggagacgtcgagtccaaccctgggcctatggttaaagttat-3′**
***2A/Ic-CCre*-R**	**5′-aagcttgagctcctacaccttcctcttcttcttgggatcgccatcttcc-3′**
**qRT-PCR**	
***qNCre*-F**	**5′ -GGATTGCTTATAACACCC -3′**
***qNCre*-R**	**5′-CGACCAGTTTAGTTACCC -3′**
***qCCre*-F**	**5′ -GCTAAGGATGACTCTGGT -3′**
***qCCre*-R**	**5′ -TTCACTATCCAGGTTACG -3′**
***qGFP*-F**	**5′-CATGCCTGAGGGATACGTG-3′**
***qGFP*-R**	**5' -GATGTTGTGGCGGGTCTT-3'**
***qGUS*-F**	**5′ -GAGCACAAGGGCGGATTC-3′**
***qGUS*-R**	**5′ -AACGGGGTCGTGTAGATTTT-3′**
***qBAR*-F**	**5′ -AACTTCCGTACCGAGCCG-3′**
***qBAR*-R**	**5′ -CCCGATGACAGCGACCAC-3′**
***qNPTII*-F**	**5′ -TGGGCACAACAGACAATCG-3′**
***qNPTII*-R**	**5′-TAGCAGCCAGTCCCTTCC-3′**
***qUBQ*-F**	**5′-CCCTAACGGGAAAGACGAT-3′**
***qUBQ*-R**	**5′-AGAACAAGATGAAGGGTGGACT-3′**
