# Supplementary material for: Dual Reproductive Cell-Specific Promoter-Mediated Split-Cre/LoxP System Suitable for Exogenous Gene Deletion in Hybrid Progeny of Transgenic Arabidopsis
Source: Int J Mol Sci. 2021 May 11;22(10):5080. doi: 10.3390/ijms22105080 (PMC8151399; doi:10.3390/ijms22105080)
Supplement: Supplementary file 1 [file ijms-22-05080-s001.zip › Supplementary File - revised/Table S5.pdf]

Table S5. List of hybrid combinations in this study.

proDD45-CCre×pro35S-NCre			
**Maternal lines (*pro35S-NCre*)**	L1	L1	L1
**Paternal lines (*proDD45-CCre*)**	L1	L2	L3
**F2 progeny lines**	L3	L5	L6

proACA9-CCre×pro35S-NCre			
**Maternal lines (*proACA9-CCre*)**	L1	L2	L3
**Paternal lines (*pro 35S-NCre*)**	L1	L1	L1
**F2 progeny lines**	L1	L6	L7

proDLL-CCre×pro35S-NCre			
**Maternal lines (*proDLL-CCre*)**	L1	L2	L3
**Paternal lines (*pro35S-NCre*)**	L1	L1	L1
**F2 progeny lines**	L3	L7	L14

proACA9-CCre×proDD45-NCre									
**Maternal lines (*proACA9-CCre*)**	L1	L2	L3	L1	L2	L3	L1	L2	L3
**Paternal lines (*proDD45-NCre*)**	L10	L10	L10	L12	L12	L12	L16	L16	L16
**F2 progeny lines**	L1	L2	L3	L4	L5	L6	L7	L8	L9

proDLL-CCre×proDD45-NCre									
**Maternal lines (*proDLL-CCre*)**	L1	L2	L3	L1	L2	L3	L1	L2	L3
**Paternal lines (*proDD45-NCre*)**	L10	L10	L10	L12	L12	L12	L16	L16	L16
**F2 progeny lines**	L1	L2	L3	L4	L5	L6	L7	L8	L9
