# Supplementary figures and images for: Dual Reproductive Cell-Specific Promoter-Mediated Split-Cre/LoxP System Suitable for Exogenous Gene Deletion in Hybrid Progeny of Transgenic Arabidopsis
Source: Int J Mol Sci. 2021 May 11;22(10):5080. doi: 10.3390/ijms22105080 (PMC8151399; doi:10.3390/ijms22105080)

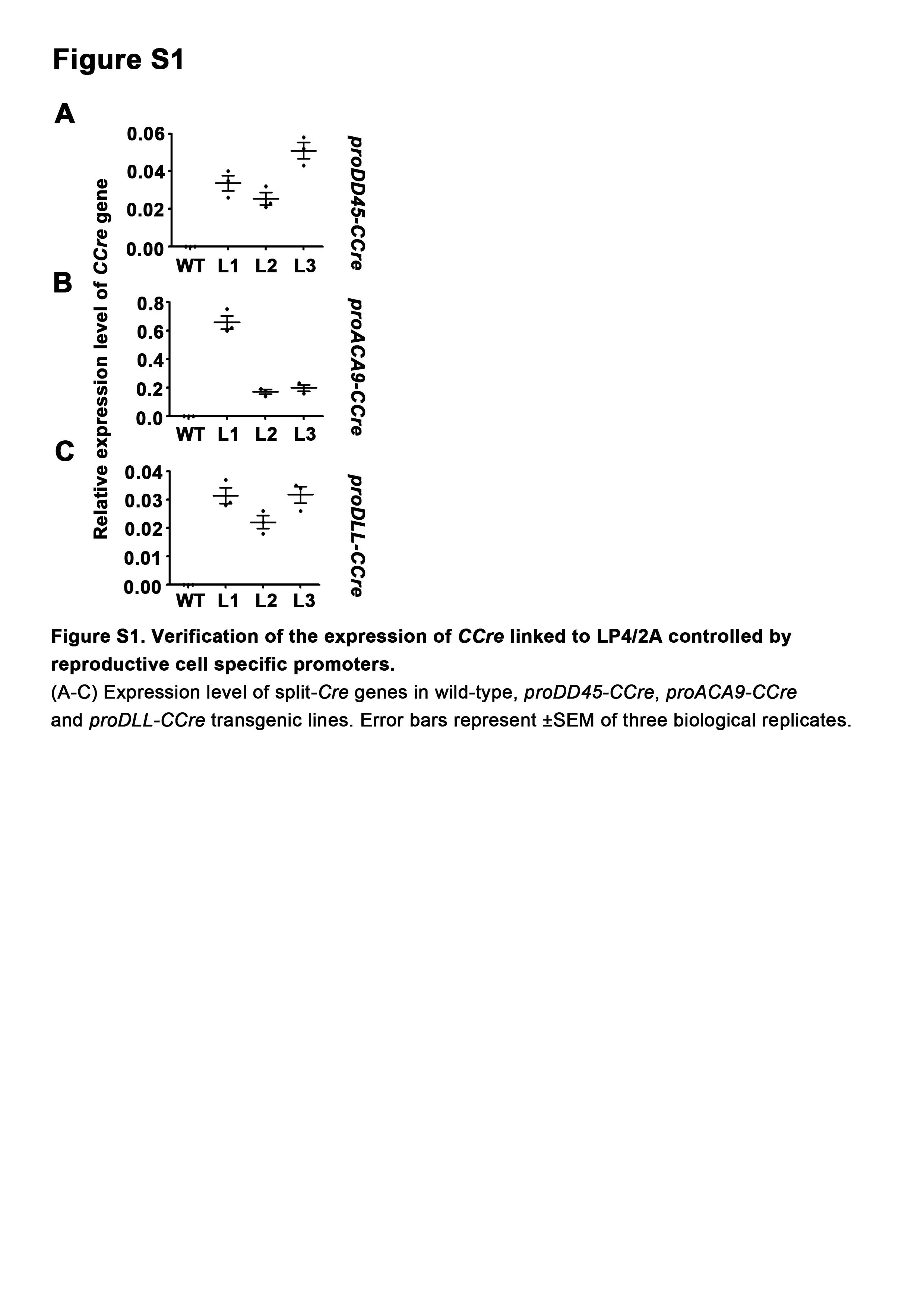

Supplement: Supplementary file 1 [file ijms-22-05080-s001.zip › Supplementary File - revised/Figure S1 - revised.jpg]

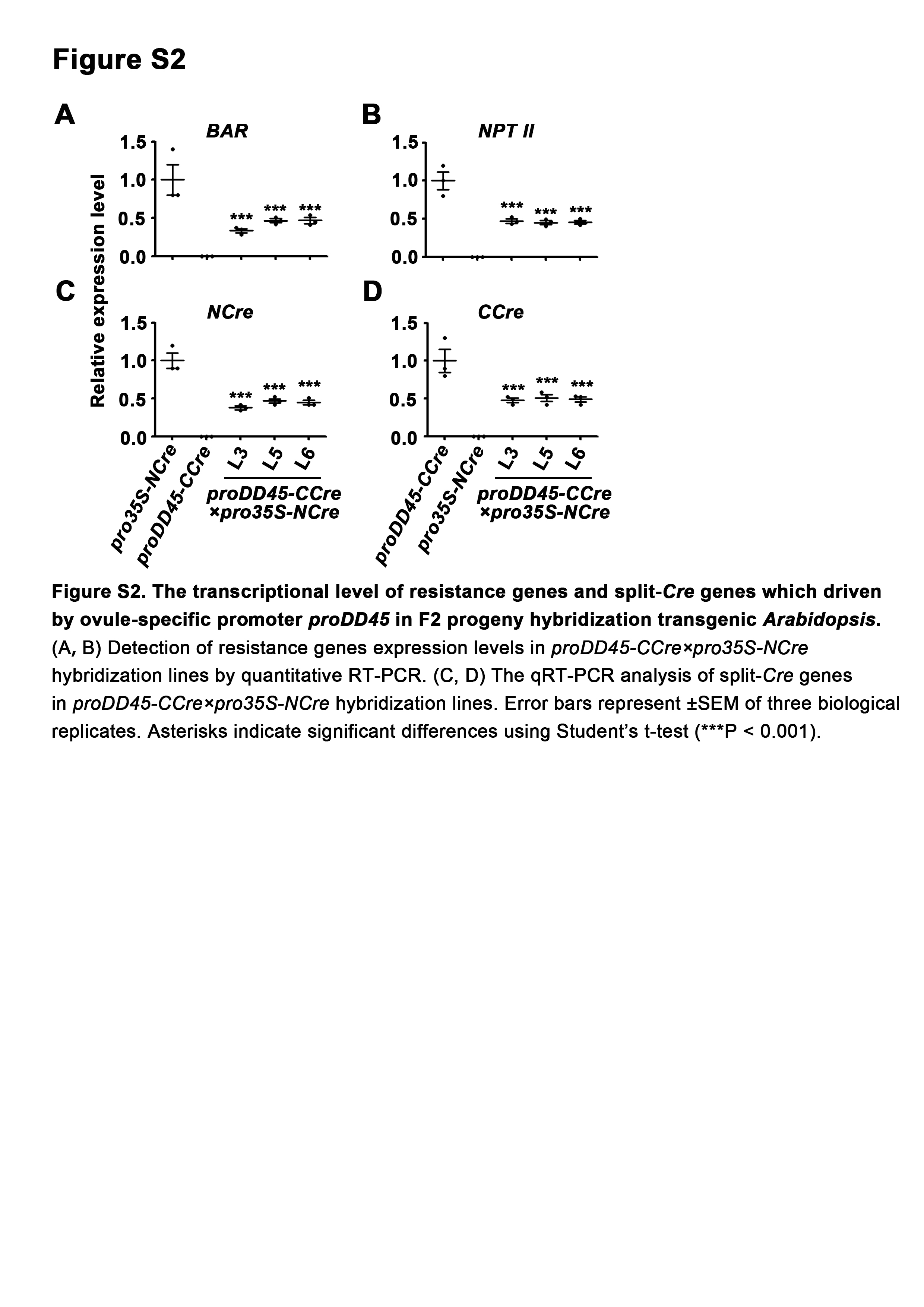

Supplement: Supplementary file 1 [file ijms-22-05080-s001.zip › Supplementary File - revised/Figure S2 - revised.jpg]

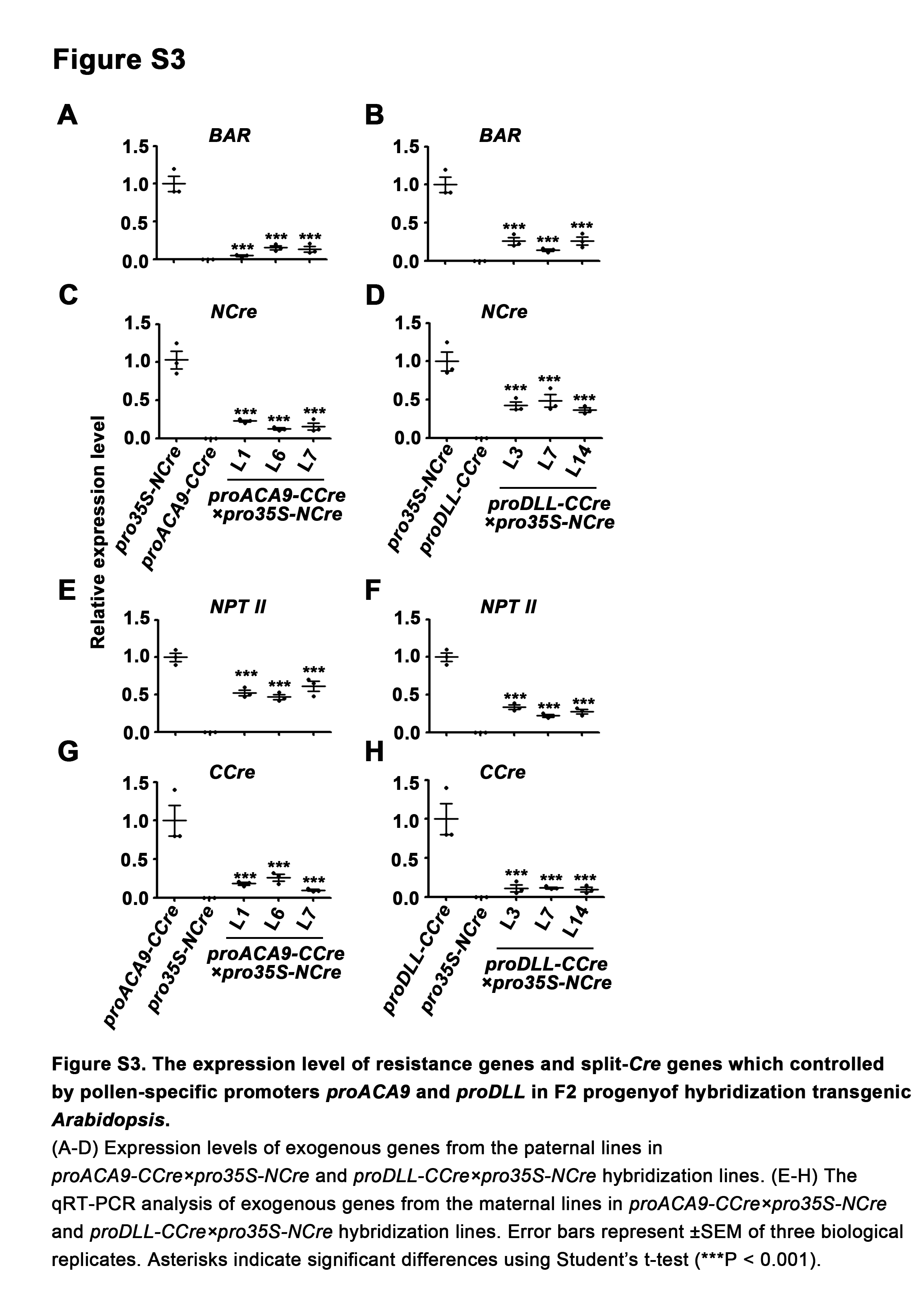

Supplement: Supplementary file 1 [file ijms-22-05080-s001.zip › Supplementary File - revised/Figure S3 - revised.jpg]

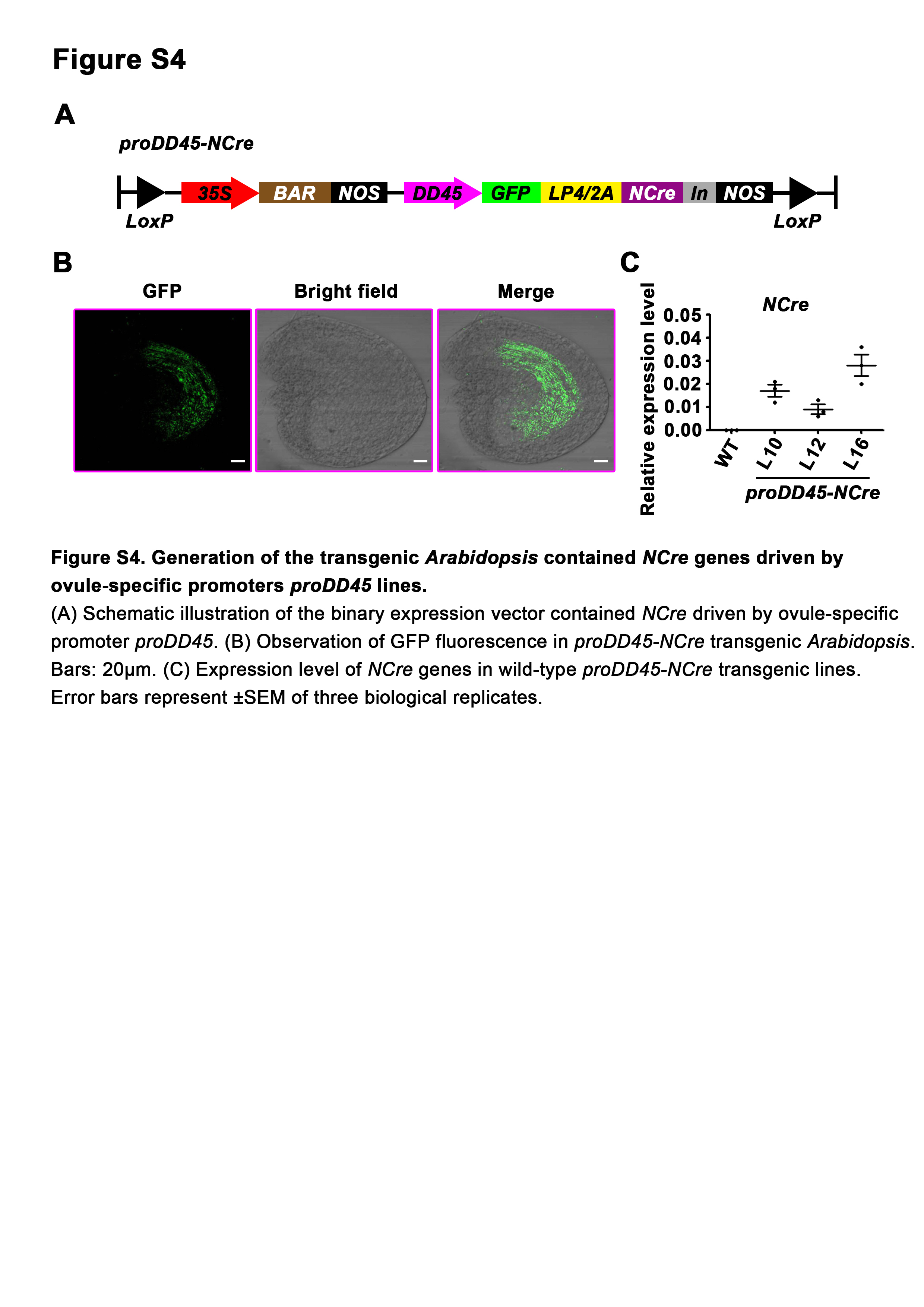

Supplement: Supplementary file 1 [file ijms-22-05080-s001.zip › Supplementary File - revised/Figure S4 - revised.jpg]

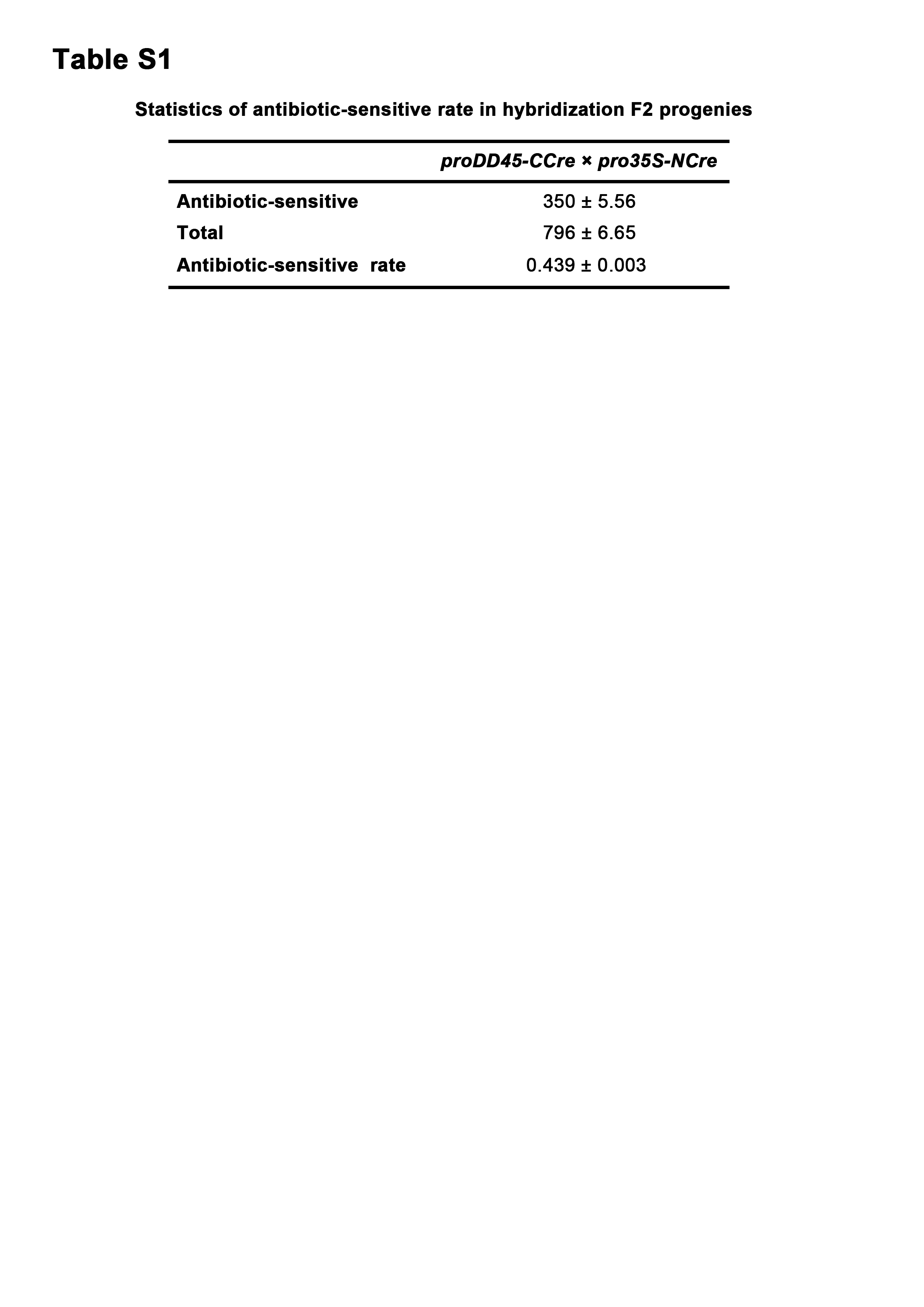

Supplement: Supplementary file 1 [file ijms-22-05080-s001.zip › Supplementary File - revised/Table S1.jpg]

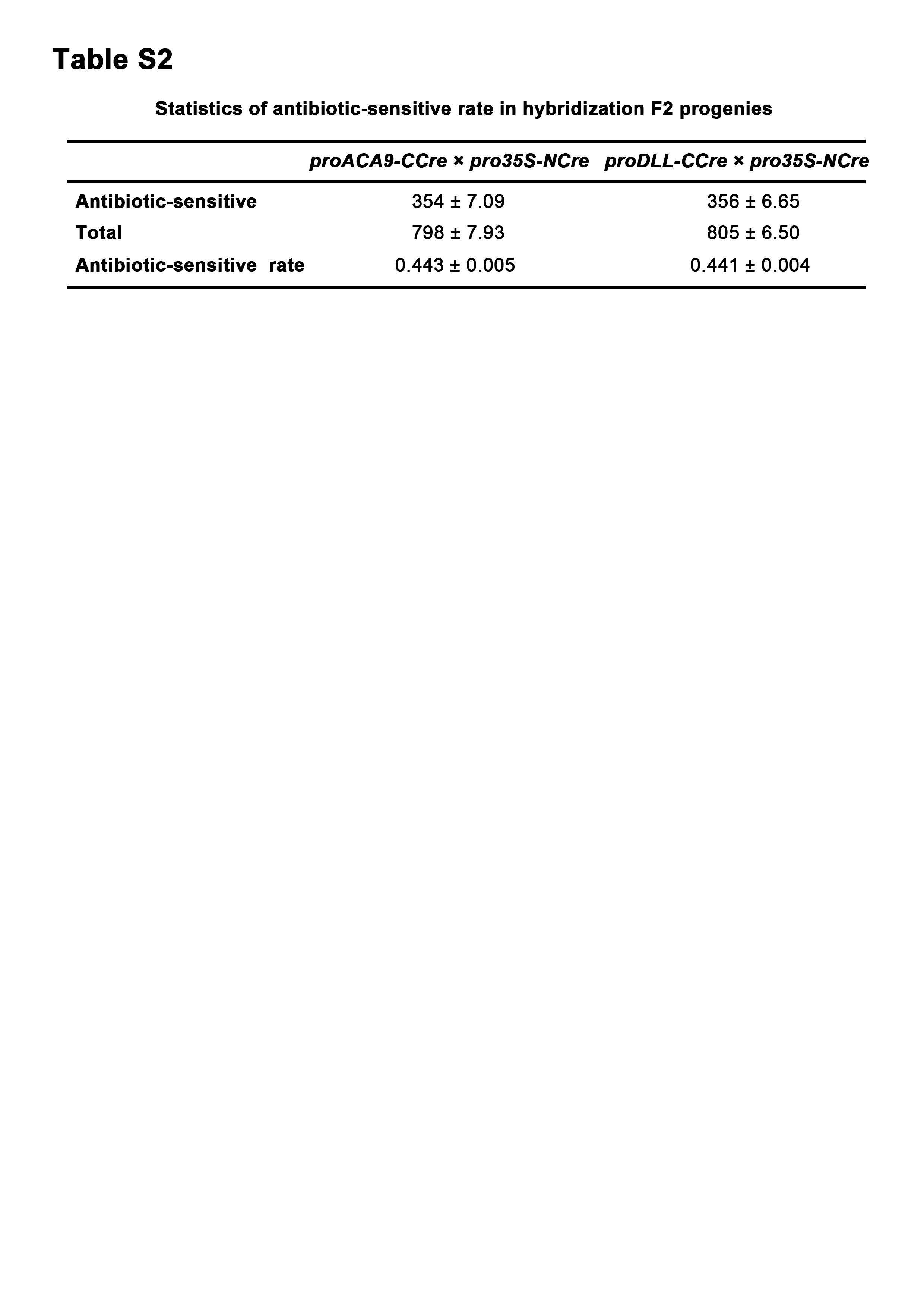

Supplement: Supplementary file 1 [file ijms-22-05080-s001.zip › Supplementary File - revised/Table S2.jpg]

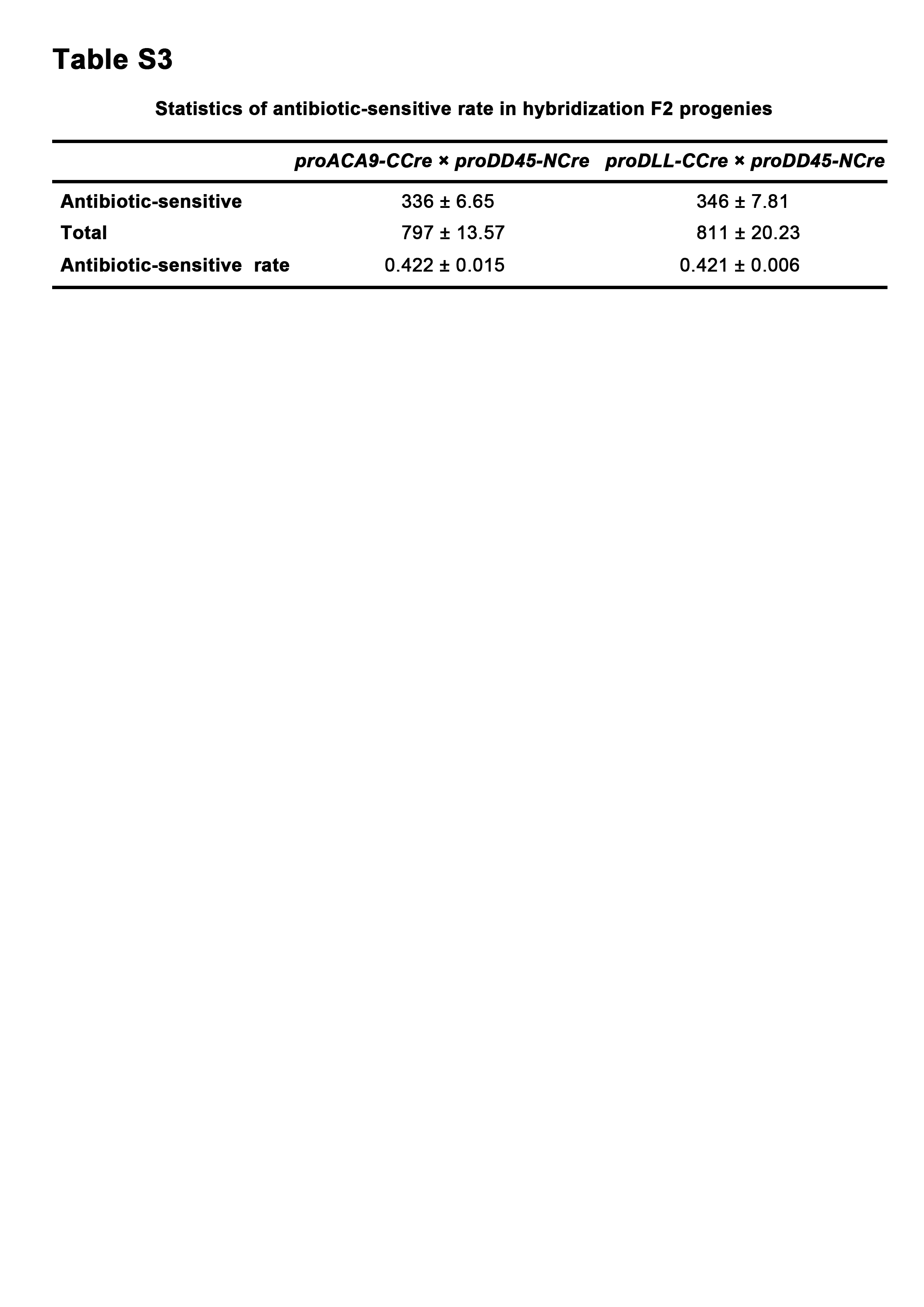

Supplement: Supplementary file 1 [file ijms-22-05080-s001.zip › Supplementary File - revised/Table S3.jpg]
